# The vaginal microbiome and the risk of preterm birth: a systematic review and network meta-analysis

**DOI:** 10.1038/s41598-022-12007-9

**Published:** 2022-05-13

**Authors:** Unnur Gudnadottir, Justine W. Debelius, Juan Du, Luisa W. Hugerth, Hanna Danielsson, Ina Schuppe-Koistinen, Emma Fransson, Nele Brusselaers

**Affiliations:** 1 Department of Microbiology, Tumor and Cell Biology (MTC), Centre for Translational Microbiome Research, Karolinska Institutet, Tomtebodavägen 16, 171 65 SolnaStockholm, Sweden; 2 Science for Life Laboratory, 171 65 Solna, Sweden; 3 Sach’s Children’s and Youth Hospital, Södersjukhuset, Stockholm, Sweden; 4 Department of Women’s and Children’s Health, Uppsala University, 751 85 Uppsala, Sweden; 5 Global Health Institute, University of Antwerp, 2610 Antwerp, Belgium; 6 Department of Head and Skin, Ghent University, 9000 Ghent, Belgium

**Keywords:** Microbiology, Microbiome, Reproductive signs and symptoms

## Abstract

Preterm birth is a major cause of neonatal morbidity and mortality worldwide. Increasing evidence links the vaginal microbiome to the risk of spontaneous preterm labour that leads to preterm birth. The aim of this systematic review and network meta-analysis was to investigate the association between the vaginal microbiome, defined as community state types (CSTs, i.e. dominance of specific lactobacilli spp, or not (low-lactobacilli)), and the risk of preterm birth. Systematic review using PubMed, Web of Science, Embase and Cochrane library was performed. Longitudinal studies using culture-independent methods categorizing the vaginal microbiome in at least three different CSTs to assess the risk of preterm birth were included. A (network) meta-analysis was conducted, presenting pooled odds ratios (OR) and 95% confidence intervals (CI); and weighted proportions and 95% CI. All 17 studies were published between 2014 and 2021 and included 38–539 pregnancies and 8–107 preterm births. Women presenting with “low-lactobacilli” vaginal microbiome were at increased risk (OR 1.69, 95% CI 1.15–2.49) for delivering preterm compared to *Lactobacillus crispatus* dominant women. Our network meta-analysis supports the microbiome being predictive of preterm birth, where low abundance of lactobacilli is associated with the highest risk, and *L. crispatus* dominance the lowest.

## Introduction

Preterm birth (< 37 completed gestation weeks), which accounts for over 10% of births worldwide, is a major cause of neonatal mortality and morbidity^1^. Many factors can trigger premature labour onset, including preterm premature rupture of membranes (PPROM), infections (e.g. *Trichomonas vaginalis* and *Chlamydia trachomatis*^2^) and microbial invasion of the amniotic cavity^3,4^. The vaginal microbiome is thought to protect from such infections, with low diversity microbiome dominated by *Lactobacillus* species considered “healthy”. In contrast, a diverse microbiome with low abundance of lactobacilli and high amounts of anaerobic bacteria can cause dysbiosis, overlapping with the clinical bacterial vaginosis (BV) diagnosis^5–7^. BV is often asymptomatic, yet has been associated with higher risks of genital infections and complications, including human papillomavirus (HPV) infections^8,9^ and pelvic inflammatory disease^10^. It has also been proposed that different *Lactobacillus* species may present different risk profiles for various adverse events^5,8^. Since vaginal dysbiosis affects millions of women, it is important to understand the role of the vaginal microbiome in preterm birth^5,11^. Currently there are few studies available that assess the relationship between the vaginal microbiome and preterm birth, with conflicting findings on whether the vaginal microbiome can influence the risk of preterm birth^2,12^.

Although meta-analyses are a great tool to pool the results from different studies, common challenges are clinical and methodological heterogeneity. Meta-analysing microbiome studies is particularly difficult because of diverse study designs, limited power and a large variety in sampling and processing techniques, including different hypervariable regions targeted^13,14^. These challenges were described in a systematic review based on culture-independent methods to assess the vaginal microbiome and preterm birth, which included nine studies^15^. One systematic review included an individual-patient meta-analysis, yet only five cohorts had sequencing data publicly available^16^.

Our group recently introduced a novel method into the microbiome meta-analysis field to assess the relationship between the vaginal microbiome and the risk of HPV infections^8^. This network meta-analysis approach is based on aggregated data; and can be used to compare different microbiome “categories” in the same statistical model, based on direct and indirect evidence. Although categorizing the vaginal microbiome has its challenges, community state types (CSTs)^6^ are commonly used and easy to interpret.

We used this network-meta-analysis method to assess the association between the vaginal microbiome (as CSTs) and the risk of preterm birth, based on a comprehensive systematic review.

## Methods

### Study selection and criteria

Only longitudinal studies were considered, in which the vaginal microbiome was assessed clearly before the onset of labour, including premature rupture of membranes and other labour-associated complications; and in which all participants were followed up until delivery. Studies exclusively including high risk pregnancies were excluded to minimize the effects of risk factors apart from the microbiome (e.g., only women with prior preterm birth, cervical weakness). Original studies were eligible if they reported the risk of preterm birth in at least three CSTs or vaginal microbiome compositions^6^, with sufficient data to report the risk per individual and not per number of samples if multiple samples were collected per woman. The earliest pregnancy samples were used for the analysis if feasible. To enable the identification of species without the need for culturing, 16S analysis of samples was preferred. Since 16S sequencing techniques have only been available recently, only studies published since 2010 were included. As this study is based on aggregated data, we used the categorization of preterm and term delivery as reported in each paper, yet if possible, the categorization of the World Health Organization was used, defining preterm birth as birth before 37 completed weeks of gestation^1^.

We excluded intervention studies, cross-sectional studies with sampling after onset of labour, studies only investigating specific pathogens or only using culture-dependent or microscopic diagnostic methods. Reviews, editorial letters, case reports, conference abstracts, books, book chapters and commentaries were also excluded. We did not use language restrictions, to minimize the risk of language bias. No restrictions were used regarding the age of the included individuals or the study setting. If two or more studies presented the same cohort or overlapping cohorts the most recent study was included or both studies were considered as one study.

All results were reported according to the Preferred Reporting Items for Systematic Reviews and meta-analysis (PRISMA) extension for network meta-analysis^17^.

### Information sources and search strategy

The search was conducted in PubMed, Web of Science, Embase and Cochrane Library and was last updated May 2021 (see search strings in Supplementary Table S1). The results were uploaded to EndNote X9 for the literature selection. The databases Prospero and Cochrane database of systematic reviews were searched to see if there were any ongoing studies on the subject.

The literature selection was conducted by two authors (UG & NB), by first removing all clearly irrelevant articles, followed by abstract and finally full text screening based on the eligibility criteria mentioned below.

### Data extraction and assessment of risk of bias

We collected the following data (if available): study characteristics (country, setting, study design and period), study population (age range, race/ethnicity, recruitment, and specific inclusion criteria), information on exposure (i.e. factors that may affect the recorded vaginal microbiome composition: gestation week of sampling, CST, method of analysis and diversity measurements), and outcome characteristics (pregnancy week of birth, spontaneous or induced birth).

The quality of included studies was assessed by a customized checklist by two authors (UG & NB) (see Supplementary Table S2).

### Data synthesis

Data used for the meta-analysis was extracted in double (UG, NB) to ensure quality, and meta-analyses were only conducted if at least three studies reported the required data.

We grouped the CSTs into five categories based on the dominating species: *L. crispatus, L. gasseri, L. iners*, “low-lactobacilli” and L. jensenii. “Low-lactobacilli” was defined as an increased diversity of anaerobic or a mixture of aerobe and facultative anaerobe bacteria (such as *Gardnerella* and *Prevotella*) based on the cut-offs and categorization used in the individual studies. CSTs which could not be transformed into these groups were omitted from the analysis. If possible, subgroup analyses were conducted based on study design, categorization of preterm birth (gestational week, spontaneous or not) and geographic region. These subgroups were chosen since spontaneous preterm birth could have different causes than induced preterm birth, and since the vaginal microbiome can differ depending on ethnicity/race^6^.

All analyses were conducted with Stata (MP 14, Stata Corporation), using the metaprop_one^18^ and network packages. The cumulative proportions of “low-lactobacilli” in each study were pooled and weighted using random effects models (to incorporate within-studies and between-studies variation)^19^, including the Freeman-Tukey double arcsine transformation to compute the weighted pooled estimate and to perform the back-transformation on the pooled estimate^18^.

To enable direct and indirect comparisons between all CSTs, we used a fixed network meta-analysis approach as described earlier^8,20^. This meta-analysis approach enables comparing different groups (CSTs) in the same statistical model in contrast to the classic pairwise meta-analysis only comparing two groups head-to-head.

A network map or network geometry^20^ was constructed to visualize all network relationships and available data on direct and indirect evidence available for the different CSTs, using crude data. The connection lines between the different dots indicate that direct information is available in at least one study, with thicker lines indicating that more studies report on this association. The larger the dots, the more studies present data on this specific CST. To assess if the results obtained by direct comparisons correspond to those obtained by indirect comparisons, the consistency of the models was measured. Large p-values (> 0.05) of the overall test and of the individual loop consistency tests imply that the consistency assumption can be accepted, and that this model can be used to give reliable assessments of the associations based on the available data. Forest plots were used to visualize and summarize the available evidence, presenting odds ratios (OR) and 95% confidence intervals (CI). In addition, the different CSTs were ranked depending on increasing risk of the outcomes, presented as relative probability which CST provides the “best outcome”, second best outcome, etc. These probability rankings should be interpreted with caution in observational settings with unbalanced groups. The number of included studies was too low for constructing funnel plots (to assess bias by small study effects).

In addition, average richness and diversity indices of each paper using either Chao1, Evenness (Simpson or Pielou) or Shannon index were pooled if sufficient data were available (at least 3 studies with means and standard deviations for both term and preterm pregnancies).

## Results

### Study selection

**Figure 1:**
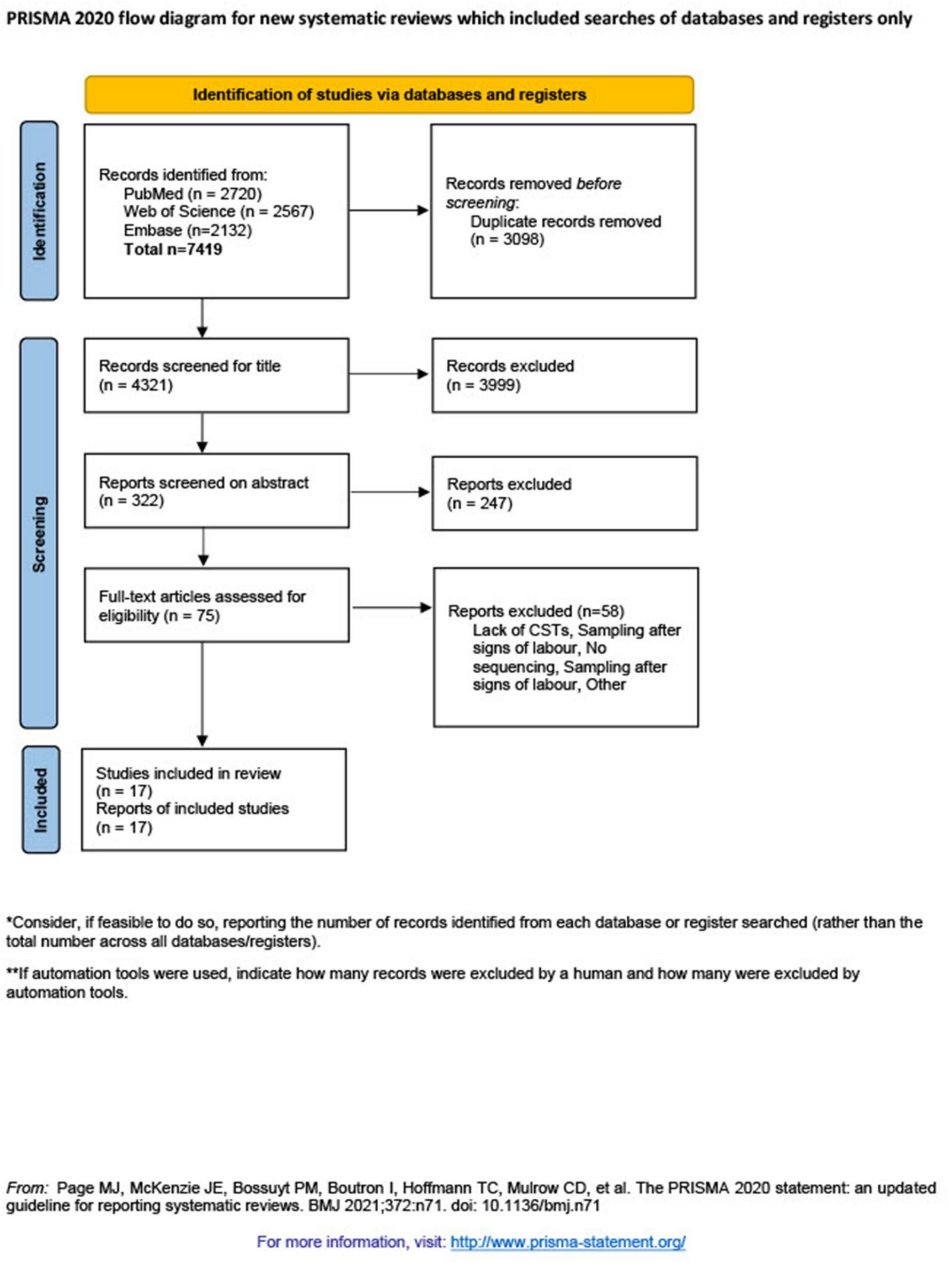
PRISMA flowchart of selection of articles included in the network meta-analysis.

Out of 4321 unique articles, 17 cohort studies were included, all published in English between 2014 and 2021 (Fig. 1). None of the 79 retrieved studies in other languages were relevant. The number of pregnancies per study ranged between 38 and 539, with 8 and 107 preterm births.

The most common exclusion criteria of otherwise eligible studies were the lack of CST grouping of results (Supplementary Table S3). In the final selection of studies, seven were excluded because a lack of CST grouping^11,21–26^, all women receiving cervical cerclage^27^, sampling after signs of labor^28^, no information regarding preterm birth for current pregnancy^29^, only the use of polymerase chain reaction (PCR) instead of sequencing^30^ or multiple CSTs assigned to each woman because of multiple sampling points^31^.

### Study characteristics and quality

Out of the 17 eligible studies, seven originated from North-America^4,5,32–36^, three from Europe^37–39^, two from South-America^40,41^, three from Asia^42–44^ and two from Africa^45,46^. Microbiome samples were taken before the third trimester in all studies. Five studies specified that women at high-risk of preterm birth were not excluded from the cohort^37–39,43,45^, while others did not specify the risk profiles. Out of the five studies that included high risk women, one study included 29 HIV positive women^45^, one included women diagnosed with preterm prelabour rupture of membranes (PPROM)^43^ and three included unspecified high-risk women^37–39^.

Preterm birth was defined as birth before 37 completed weeks of gestation for all studies except one, where it was defined as before 34 weeks of gestation^4^. In twelve studies, a healthcare professional took the samples^4,5,32,37–39,41–46^, while the other five had self-sampling^33–36,40^. Furthermore, all studies except for two reported that the onset of preterm birth was spontaneous (Supplementary Table S4)^45,46^.

All studies used 16S analysis of the microbiome samples, except one which used shotgun sequencing^37^. Among the 16S studies, four hypervariable regions of the 16S molecule were targeted, with most studies targeting either the V1-3 or V3-4 hypervariable regions (Supplementary Table S4). The studies reported up to 13 different CSTs, which were re-categorized as mentioned above. Shannon diversity index was reported in 15 out of the 17 studies^4,5,32–38,40,42–46^, but it varied if mean or median value was used, if standard deviation was reported and if the value was reported as number or as figure, so pooling was not feasible. The other diversity measures were not reported frequently enough to pool the results.

### Synthesis of results

**Figure 2:**
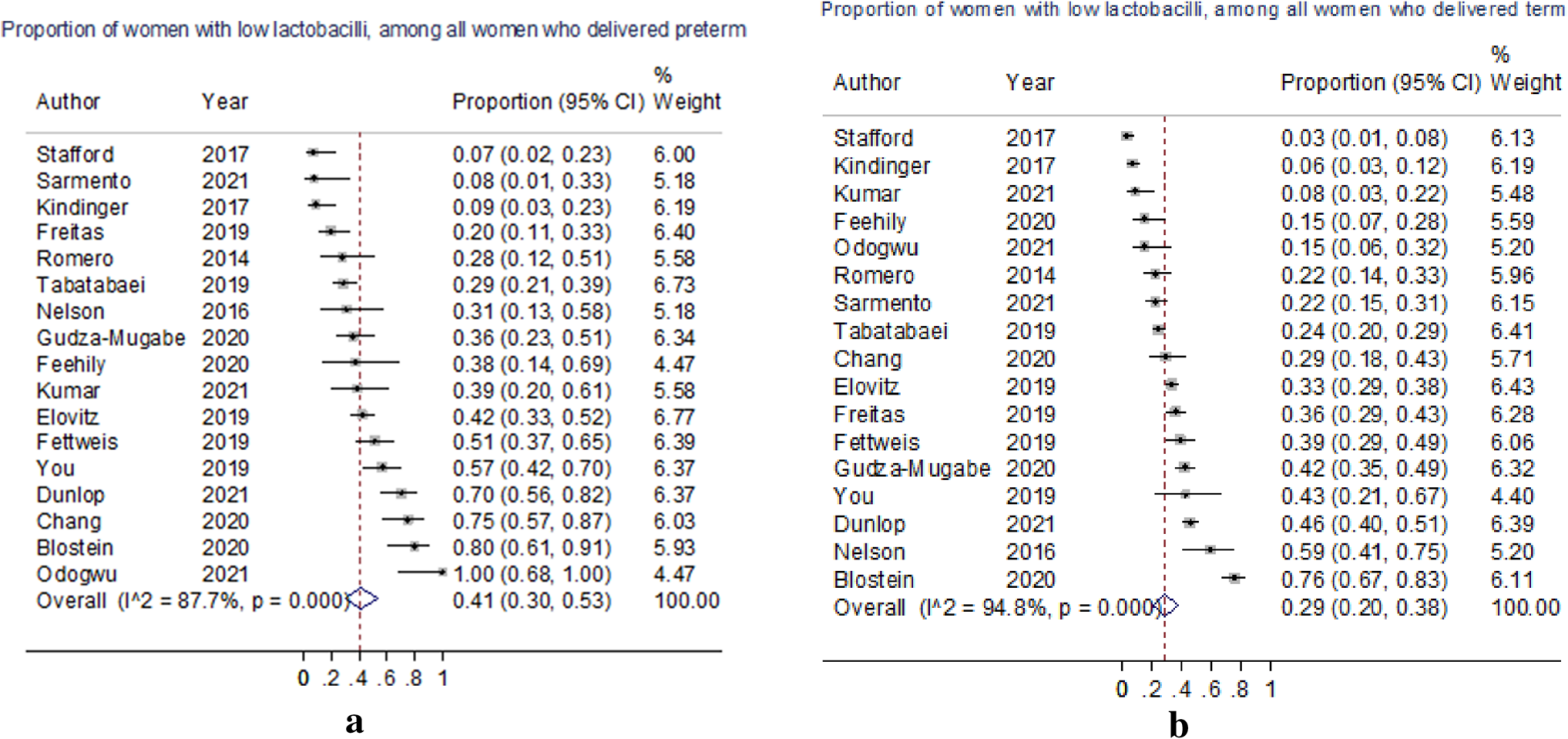
Forest plots showing all 17 included studies and the pooled and weighted proportion of “low-lactobacilli” women who delivered (**a**) preterm and (**b**) at term.

Among women who delivered preterm, the pooled proportion with “low-lactobacilli” was 0.41 (95% CI 0.30–0.53) compared to 0.29 (95% CI 0.20–0.38) of women with term deliveries (Fig. 2).

**Figure 3:**
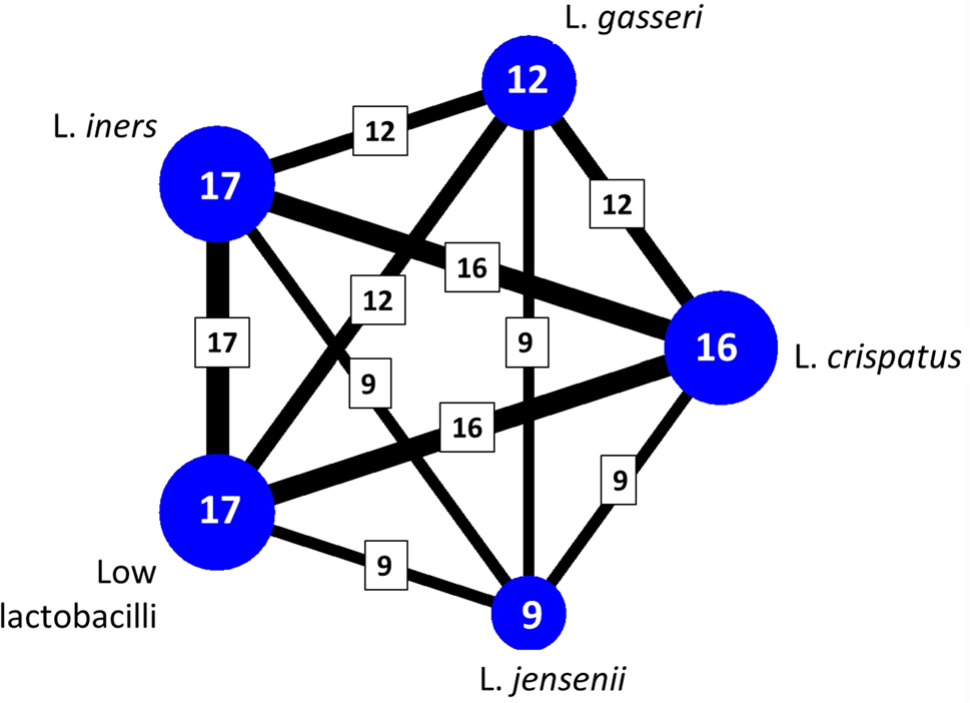
Network map of all 17 included studies by vaginal microbiome composition, showing how many studies reported which community state types (CSTs). Legend: Each dot represents a CST, with the number indicating how many studies reported it. The lines between the blue dots and their thickness represent the number of studies which reported the CSTs joined by the line.

The network map (Fig. 3) indicates that direct evidence was available for the association between all five CST categories (at least eight studies reported on each CST category). The test for inconsistency indicated overall consistency (p = 0.77), and so did all loop inconsistency tests (p > 0.05), indicating this method can be used to assess the associations between the different CSTs.

**Figure 4:**
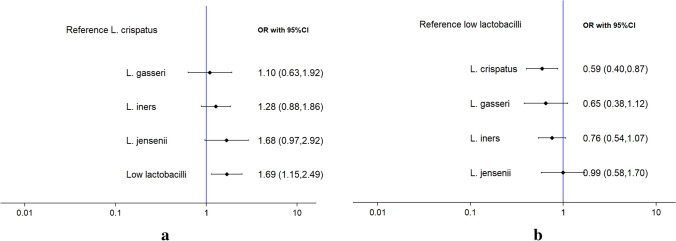
Forest plots comparing community state types (CSTs) and their risk of preterm birth using (**a**) *Lactobacillus crispatus* and (**b**) “Low-lactobacilli” as reference group, where an odds ratio (OR) > 1.00 indicates association with preterm birth.

The risk of preterm birth was higher among women presenting with “low-lactobacilli” compared to *L. crispatus* (OR 1.69, 95% CI 1.15–2.49) (Fig. 4). The risk of preterm birth was also high among women with *L. jensenii* compared to *L. crispatus* (OR 1.68, 95% CI 0.97–2.92), yet these results did not reach statistical significance.

Ranking tests showed that the *L. crispatus* dominant group was most probable to be the “best” microbiome composition, and L. jensenii the most probably the “worst” group considering the association with preterm birth (figure not shown).

### Subgroup analyses

Four different subgroup analyses were conducted: (1) Preterm birth defined < 37 weeks (excluding the one study only including early preterm birth)^5,32–34,36–46^, (2) Clear spontaneous preterm birth^4,5,32–34,36–44^, (3) Region North-America and Europe^4,5,32–39^ and (4) Region South-America, Asia and Africa^40–46^ (Table 1). These subgroup analyses showed consistent results with the overall analysis, although the analyses for preterm birth as < 37 weeks had insufficient power (Table 1).

**Table 1:** Subgroup analysis by definitions of preterm birth, geographical region and if all cases of preterm birth were clearly spontaneous.

Subgroup	Number of deliveries		Proportion "Low-lactobacilli"				Odds ratio (95% confidence interval)						
	N preterm	N term	% among preterm (95% confidence interval)	% among term (95% confidence interval)	p-value overall consistency	p-values "loop inconsistencies"	L. crispatus (reference)	L. gasseri	L. iners	“Low-lactobacilli”	L. jensenii	Studies included	N studies
Overall	570	1962	0.41 (0.30, 0.53)	0.29 (0.20, 0.38)	0.7739	all > 0.05	1.00	1.10 (0.63, 1.92)	1.28 (0.88,1.86)	**1.69 (1.15, 2.49)**	1.68 (0.97, 2.92)	^4, 5, 32–46^	17
Preterm < 37 weeks	552	1890	0.42 (0.30, 0.55)	0.29 (0.20, 0.39)	0.6065	Some loop inconsistencies*	1.00	1.13 (0.64, 2.01)	1.33 (0.90, 1.98)	**1.76 (1.16, 2.65)**	1.73 (0.98, 3.07)	^5, 32–46^	16
Clear spontaneous preterm birth	520	1733	0.38 (0.26, 0.51)	0.29 (0.20, 0.39)	0.9528	all > 0.05	1.00	1.17 (0.67, 2.04)	1.37 (0.94, 2.01)	**1.76 (1.19, 2.61)**	1.68 (0.96, 2.95)	^4, 5, 32–44^	15
Europe and N-America	392	1422	0.31 (0.19, 0.44)	0.26 (0.17, 0.36)	0.6524	all > 0.05	1.00	1.08 (0.63, 1.87)	1.29 (0.86, 1.94)	**1.53 (1.03, 2.26)**	1.55 (0.90, 2.67)	^4, 5, 32–39^	10
Africa, Asia and S-America	178	540	0.57 (0.36, 0.77)	0.33 (0.16, 0.52)	0.8053	all > 0.05	1.00	0.77 (0.09, 6.38)	1.11 (0.45, 2.78)	2.17 (0.86, 5.44)	2.67 (0.29, 24.83)	^40–46^	7

*Results comparing “low-lactobacilli” to other lactobacilli may be less reliable in the network meta-analysis (low power).

## Discussion

This network meta-analysis suggests that women with a “low-lactobacilli” vaginal microbiome composition were at higher risk of preterm birth (spontaneous and overall) compared to women with *L. crispatus* dominant microbiome compositions.

Our systematic review and network meta-analysis is the first of its kind, since only one meta-analysis had previously been done on this subject, which used individual level sequencing data^16^. We chose CSTs over individual sequencing data because there can be a lack of open access to the data leading to selection bias of studies, and updating the recently published individual patient data meta-analysis would not have contributed any new information to the field. Furthermore, CSTs are more complimentary to the current knowledge, and although not ideal, are good for clinical uses and for identifying targets for future developments. Therefore, we see both meta-analysis approaches as complementary. Although we also had to exclude six otherwise eligible studies because CSTs were not reported, we were able to include 17 studies, compared to the six studies of the previous meta-analysis^16^ (only one study in common^4^). Authors of the excluded papers were contacted for data but never replied. As mentioned above, heterogeneity of methods may propose problems and decrease the number of studies which can be included in individual patient data meta-analyses. Nonetheless, by using CST categorization we were able to compare five different groups (CSTs) to each other in this network approach, instead of just using two groups as is common in classic meta-analyses. The use of CSTs was first described in a small cohort study from 2010^6^ and has been widely used despite its challenges and limitations^47,48^, but it is currently the best option in the field to categorize vaginal microbiome compositions. Many of the included studies used an adapted form of the original CSTs, using a range from 3 to 13 groups. Most common were subgroups of the diverse non-lactobacilli dominant group, but there was not enough uniformity between those in the studies to use subgrouping for this analysis. Despite these factors, our inconsistency tests gave robust results showing that the method is stable enough to use the results.

When comparing microbiome studies, there are always many factors that can influence the results, such as the sample collection, extraction methods and hypervariable region used when sequencing^13^. The motivation for hypervariable region selection varies, with emphasis placed on universality or specificity. The V4 hypervariable region is more conserved among *Lactobacillus* species, making species-level assignments more uncertain. In contrast, the long amplicons generated by the V1-V3 hypervariable region can be challenging for short-read technologies^49^.

Different studies may also have different cut-off levels for what defines a “dominant” group, and that cut-off is seldom specified. Furthermore, in the included studies the social or physical underlying risk of preterm birth of the women was not always well-defined or incorporated in the analyses.

Our results are consistent with the previously conducted meta-analysis^16^, which showed that women that delivered preterm had higher diversity in their vaginal microbiome, which is associated with the “low-lactobacilli” CST. The reviews on this subject also agree that even though current studies are not all consistent, it seems that overall *L. crispatus* is protective and a “low-lactobacilli” microbiome might increase the risk of spontaneous preterm birth^2,12,15^. Furthermore, as many of the included studies were published after the systematic-review of the topic^15^, better methods and technologies might account for why more studies are now finding association between the microbiome and PTB. Yet, several of the included studies were still hampered by low sample sizes, methodological heterogeneity and selection bias (as seen by the unrepresentatively high proportions of preterm birth cases in some studies).

The “low-lactobacilli” group includes bacterial species such as *Gardnerella* and *Prevotella*, both of which are known to promote proinflammatory cytokines and are commonly found in the vaginal microbiome just before PPROM^12^. It is therefore not surprising that they may have an impact on the maternal immune response and play a part in inducing preterm birth.

These results are important for the possible prediction and prevention of preterm birth which remains an important problem today. Yet, further longitudinal studies are needed to incorporate potential natural changes in the vaginal microbiome during pregnancy^4^, and to better understand the pathophysiological mechanisms underlying these apparent different risk profiles.

## Conclusion

To conclude, the diversity of the vaginal microbiome seems to play a part in the risk of preterm birth, where women with low abundance of lactobacilli were at greater risk of delivering preterm compared to women with *L. crispatus* dominant microbiome.

## Supplementary Information

Supplementary Information 1.

Supplementary Information 2.

## Data Availability

All data is available from the included articles and in the Supplement.

## References

[1] (WHO) WHO. *Preterm Birth*. https://www.who.int/news-room/fact-sheets/detail/preterm-birth.

[2] Bayar E, Bennett PR, Chan D, Sykes L, MacIntyre DA (2020). The pregnancy microbiome and preterm birth. Semin. Immunopathol..

[3] Baldwin EA, Walther-Antonio M, MacLean AM, Gohl DM, Beckman KB, Chen J (2015). Persistent microbial dysbiosis in preterm premature rupture of membranes from onset until delivery. PeerJ.

[4] Romero R, Hassan SS, Gajer P, Tarca AL, Fadrosh DW, Bieda J (2014). The vaginal microbiota of pregnant women who subsequently have spontaneous preterm labor and delivery and those with a normal delivery at term. Microbiome.

[5] Fettweis JM, Serrano MG, Brooks JP, Edwards DJ, Girerd PH, Parikh HI (2019). The vaginal microbiome and preterm birth. Nat. Med..

[6] Ravel J, Gajer P, Abdo Z, Schneider GM, Koenig SS, McCulle SL (2011). Vaginal microbiome of reproductive-age women. Proc. Natl. Acad. Sci. USA.

[7] van de Wijgert JH, Borgdorff H, Verhelst R, Crucitti T, Francis S, Verstraelen H (2014). The vaginal microbiota: What have we learned after a decade of molecular characterization?. PLoS ONE.

[8] Norenhag J, Du J, Olovsson M, Verstraelen H, Engstrand L, Brusselaers N (2020). The vaginal microbiota, human papillomavirus and cervical dysplasia: A systematic review and network meta-analysis. BJOG.

[9] Brusselaers N, Shrestha S, van de Wijgert J, Verstraelen H (2019). Vaginal dysbiosis and the risk of human papillomavirus and cervical cancer: Systematic review and meta-analysis. Am. J. Obstet. Gynecol..

[10] Chen X, Lu Y, Chen T, Li R (2021). The female vaginal microbiome in health and bacterial vaginosis. Front. Cell. Infect. Microbiol..

[11] Hyman RW, Fukushima M, Jiang H, Fung E, Rand L, Johnson B (2014). Diversity of the Vaginal Microbiome Correlates With Preterm Birth. Reprod Sci..

[12] Tsonis O, Gkrozou F, Harrison E, Stefanidis K, Vrachnis N, Paschopoulos M (2020). Female genital tract microbiota affecting the risk of preterm birth: What do we know so far? A review. Eur. J. Obstet. Gynecol. Reprod. Biol..

[13] Hugerth LW, Andersson AF (2017). Analysing microbial community composition through amplicon sequencing: From sampling to hypothesis testing. Front. Microbiol..

[14] Debelius J, Song SJ, Vazquez-Baeza Y, Xu ZZ, Gonzalez A, Knight R (2016). Tiny microbes, enormous impacts: What matters in gut microbiome studies?. Genome Biol..

[15] Peelen M, Luef BM, Lamont RF, de Milliano I, Jensen JS, Limpens J (2019). The influence of the vaginal microbiota on preterm birth: A systematic review and recommendations for a minimum dataset for future research. Placenta.

[16] Kosti I, Lyalina S, Pollard KS, Butte AJ, Sirota M (2020). Meta-analysis of vaginal microbiome data provides new insights into preterm birth. Front. Microbiol..

[17] Hutton B, Salanti G, Caldwell DM, Chaimani A, Schmid CH, Cameron C (2015). The PRISMA extension statement for reporting of systematic reviews incorporating network meta-analyses of health care interventions: Checklist and explanations. Ann. Intern. Med..

[18] Nyaga VN, Arbyn M, Aerts M (2014). Metaprop: A Stata command to perform meta-analysis of binomial data. Arch. Public Health..

[19] Michael Borenstein L. H. *Hannah Rothstein Meta-Analysis Fixed effect vs. random effects 2007*. https://www.meta-analysis.com/downloads/M-a_f_e_v_r_e_sv.pdf.

[20] Shim S, Yoon BH, Shin IS, Bae JM (2017). Network meta-analysis: Application and practice using Stata. Epidemiol. Health..

[21] Nasir SA, Mohammed BK, Alabsi SGJ (2018). Lactobacillus species detected by 16S rRNA gene sequence isolated from the vaginae of pregnant women and its relation to preterm labor. Int. J. Res. Pharm. Sci..

[22] Stout MJ, Zhou YJ, Wylie KM, Tarr PI, Macones GA, Tuuli MG (2017). Early pregnancy vaginal microbiome trends and preterm birth. Am. J. Obstet. Gynecol..

[23] Subramaniam A, Kumar R, Cliver SP, Zhi DG, Szychowski JM, Abramovici A (2016). Vaginal microbiota in pregnancy: Evaluation based on vaginal flora, birth outcome, and race. Am. J. Perinatol..

[24] Wheeler S, Pryor K, Antczak B, Truong T, Murtha A, Seed P (2018). The relationship of cervical microbiota diversity with race and disparities in preterm birth. J. Neonatal-Perinatal Med..

[25] Callahan BJ, DiGiulio DB, Goltsman DSA, Sun CL, Costello EK, Jeganathan P (2017). Replication and refinement of a vaginal microbial signature of preterm birth in two racially distinct cohorts of US women. Proc. Natl. Acad. Sci. U.S.A..

[26] de Freitas AS, Dobbler PCT, Mai V, Procianoy RS, Silveira RC, Corso AL (2020). Defining microbial biomarkers for risk of preterm labor. Braz. J. Microbiol..

[27] Kindinger LM, MacIntyre DA, Lee YS, Marchesi JR, Smith A, McDonald JAK (2016). Relationship between vaginal microbial dysbiosis, inflammation, and pregnancy outcomes in cervical cerclage. Sci. Transl. Med..

[28] Hocevar K, Maver A, Simic MV, Hodzic A, Haslberger A, Sersen TP (2019). Vaginal microbiome signature is associated with spontaneous preterm delivery. Front. Med..

[29] Nasioudis D, Forney LJ, Schneider GM, Gliniewicz K, France M, Boester A (2017). Influence of pregnancy history on the vaginal microbiome of pregnant women in their first trimester. Sci Rep..

[30] Amabebe E, Chapman DR, Stern VL, Stafford G, Anumba DOC (2018). Mid-gestational changes in cervicovaginal fluid cytokine levels in asymptomatic pregnant women are predictive markers of inflammation-associated spontaneous preterm birth. J. Reprod. Immunol..

[31] DiGiulio DB, Callahan BJ, McMurdie PJ, Costello EK, Lyell DJ, Robaczewska A (2015). Temporal and spatial variation of the human microbiota during pregnancy. Proc. Natl. Acad. Sci. U.S.A..

[32] Freitas AC, Bocking A, Hill JE, Money DM (2018). Increased richness and diversity of the vaginal microbiota and spontaneous preterm birth. Microbiome.

[33] Nelson DB, Shin H, Wu JW, Dominguez-Bello MG (2016). The gestational vaginal microbiome and spontaneous preterm birth among nulliparous African American women. Am. J. Perinatol..

[34] Tabatabaei N, Eren AM, Barreiro LB, Yotova V, Dumaine A, Allard C (2019). Vaginal microbiome in early pregnancy and subsequent risk of spontaneous preterm birth: A case-control study. BJOG.

[35] Dunlop AL, Satten GA, Hu YJ, Knight AK, Hill CC, Wright ML (2021). Vaginal microbiome composition in early pregnancy and risk of spontaneous preterm and early term birth among African American women. Front. Cell Infect. Microbiol..

[36] Elovitz MA, Brown AG, Ravel J (2019). The cervicovaginal metabolomic signature is different among women with CST IV in the 2nd trimester who ultimately have a preterm birth. Reprod. Sci..

[37] Feehily C, Crosby D, Walsh CJ, Lawton EM, Higgins S, McAuliffe FM (2020). Shotgun sequencing of the vaginal microbiome reveals both a species and functional potential signature of preterm birth. NPJ Biofilms Microbiomes.

[38] Kindinger LM, Bennett PR, Lee YS, Marchesi JR, Smith A, Cacciatore S (2017). The interaction between vaginal microbiota, cervical length, and vaginal progesterone treatment for preterm birth risk. Microbiome.

[39] Stafford GP, Parker JL, Amabebe E, Kistler J, Reynolds S, Stern V (2017). Spontaneous preterm birth is associated with differential expression of vaginal metabolites by lactobacilli-dominated microflora. Front. Physiol..

[40] Blostein F, Gelaye B, Sanchez SE, Williams MA, Foxman B (2020). Vaginal microbiome diversity and preterm birth: Results of a nested case-control study in Peru. Ann. Epidemiol..

[41] Sarmento SGP, Moron AF, Forney LJ, Hatanaka AR, Carvalho FHC, Franca MS (2021). An exploratory study of associations with spontaneous preterm birth in primigravid pregnant women with a normal cervical length. J. Matern.-Fetal Neonatal Med..

[42] Chang DH, Shin J, Rhee MS, Park KR, Cho BK, Lee SK (2020). Vaginal microbiota profiles of native Korean Women and associations with high-risk pregnancy. J. Microbiol. Biotechnol..

[43] You YA, Kwon EJ, Choi SJ, Hwang HS, Choi SK, Lee SM (2019). Vaginal microbiome profiles of pregnant women in Korea using a 16S metagenomics approach. Am. J. Reprod. Immunol..

[44] Kumar M, Murugesan S, Singh P, Saadaoui M, Elhag DA, Terranegra A (2021). Vaginal microbiota and cytokine levels predict preterm delivery in Asian women. Front. Cell Infect. Microbiol..

[45] Gudza-Mugabe M, Havyarimana E, Jaumdally S, Garson KL, Lennard K, Tarupiwa A (2020). Human immunodeficiency virus infection is associated with preterm delivery independent of vaginal microbiota in pregnant African women. J. Infect. Dis..

[46] Odogwu NM, Chen J, Onebunne CA, Jeraldo P, Yang L, Johnson S (2021). Predominance of atopobium vaginae at midtrimester: A potential indicator of preterm birth risk in a Nigerian cohort. mSphere.

[47] France MT, Ma B, Gajer P, Brown S, Humphrys MS, Holm JB (2020). VALENCIA: A nearest centroid classification method for vaginal microbial communities based on composition. Microbiome.

[48] Ma ZS, Li L (2017). Quantifying the human vaginal community state types (CSTs) with the species specificity index. PeerJ.

[49] Klindworth A, Pruesse E, Schweer T, Peplies J, Quast C, Horn M (2013). Evaluation of general 16S ribosomal RNA gene PCR primers for classical and next-generation sequencing-based diversity studies. Nucleic Acids Res..

