# Supplementary material for: The vaginal microbiome and the risk of preterm birth: a systematic review and network meta-analysis
Source: Sci Rep. 2022 May 13;12:7926. doi: 10.1038/s41598-022-12007-9 (PMC9106729; doi:10.1038/s41598-022-12007-9)
Supplement: Supplementary file 1 — Supplementary Information 1. [file 41598_2022_12007_MOESM1_ESM.docx]

**Supplementary material**

Table S1: Search strings and databases used for the systematic review.

| **Database** | **Search string** | **Details** | **Date of most recent search** | **Number of hits** |
| --- | --- | --- | --- | --- |
| PubMed | (microbiome [tiab] OR microbiota [tiab] OR "Microbiota"[Mesh] OR 16s [tiab] OR “shot gun” [tiab] OR “community state types” [tiab] OR sequencing [tiab] OR "Sequence Analysis"[Mesh] OR dysbiosis OR "Dysbiosis"[Mesh] OR dysbiosis [tiab] OR vaginosis [tiab] OR "Vaginosis, Bacterial"[Mesh] OR vaginitis [tiab]) AND (preterm [tiab] OR pre-term [tiab] OR premature [tiab] OR "Premature Birth"[Mesh] OR prematurity[tiab] ) AND (pregnan* [tiab] OR gestation* [tiab] OR mother* [tiab] OR matern*[tiab] OR "Pregnancy"[Mesh] OR birth [tiab]) |  | 2021-05-11 | 2720 |
| Web of Science | (microbiome OR microbiota OR 16s OR “shot gun” OR “community state types” OR dysbiosis OR vaginosis OR vaginitis) AND (preterm OR pre-term OR premature OR prematurity) AND (pregnant OR gestation OR gestational OR mother OR mothers OR maternal OR pregnancy OR birth) | Search on topic | 2021-05-11 | 2567 |
| Embase | (microbiome:ab,ti OR microbiota:ab,ti OR 16s:ab,ti OR 'shot gun':ab,ti OR 'community state types':ab,ti OR dysbiosis:ab,ti OR vaginosis:ab,ti OR vaginitis:ab,ti) AND (preterm:ab,ti OR 'pre term':ab,ti OR premature:ab,ti OR prematurity:ab,ti) AND (pregnant:ab,ti OR gestation:ab,ti OR gestational:ab,ti OR mother:ab,ti OR mothers:ab,ti OR maternal:ab,ti OR pregnancy:ab,ti OR birth:ab,ti) |  | 2021-05-11 | 2132 |

Table S2: Quality assessment of the included studies

| **Author, year** | **Journal** | **Definition of PTB <37 weeks** | **Home sampling (0)/Clinician sampling (1)** | **Clear location of the vagina sampled** | **Dry tubes (0)/ buffer (1)** | **16S** | **Shannon index** | **Single test (0)/ Multiple tests (1)** | **# of CSTs** | **Hypervariable region** |
| --- | --- | --- | --- | --- | --- | --- | --- | --- | --- | --- |
| Blostein, 2020 | Annals of Epidemiology | 1 | 0 | 0 | 0 | 1 | 1 | 0 | 3 | V4 |
| Chang, 2020 | JMB | 1 | 1 | 0 | 0 | 1 | 1 | 0 | 3 | V1 - V3 |
| Dunlop, 2021 | Frontiers in cellular and infection microbiology | 1 | 0 | 1 | 0 | 1 | 1 | 0 | 8 | V3 – V4 |
| Elovitz, 2019 | Nature Communications | 1 | 0 & 1 | 1 | 1 | 1 | 1 | 1 | 6 | V3-V4 |
| Feehily, 2020 | npj Biofilms and Microbiomes | 1 | 1 | 1 | Not available | 0 | 1 | 0 | 6 | Shotgun sequencing |
| Fettweis, 2019 | Nature Medicine | 1 | 1 | 1 | 1 | 1 | 1 | 1 | 13 | V1 - V3 |
| Freitas, 2018 | Microbiome | 1 | 1 | 0 | 0 | 1 | 1 | 0 | 7 | V3 |
| Gudza-Mugabe, 2020 | The Journal of infectious diseases | 1 | 1 | 0 | 1 | 1 | 1 | 0 | 5 | V4 |
| Kindinger, 2017 | Microbiome | 1 | 1 | 1 | 0 | 1 | 1 | 0 & 1 | 5 | V1 - V3 |
| Kumar, 2021 | Frontiers in cellular and infection microbiology | 1 | 1 | 1 | 0 | 1 | 1 | 1 | 5 | V1-V3 |
| Nelson, 2016 | Am J Perinatol | 1 | 0 | 0 | 0 | 1 | 1 | 0 | 5 | V4 |
| Odogwu, 2021 | American Society of Microbiology | 1 | 1 | 1 | 1 | 1 | 1 | 0 | 5 | V3-V5 |
| Romero, 2014 | Microbiome | 0 | 1 | 1 | 0 | 1 | 1 | 1 | 3 | V1 - V3 |
| Sarmento, 2021 | The J of Maternal-Fetal & Neonatal Med | 1 | 1 | 1 | 0 | 1 | 0 | 0 | 8 | V1-V3 |
| Stafford, 2017 | Frontiers in Physiology | 1 | 1 | 1 | 0 | 1 | 0 | 0 & 1 | 5 | V1 - V3 |
| Tabatabaei, 2019 | BJOG | 1 | 0 | 0 | 0 | 1 | 1 | 0 | 6 | V4 |
| You, 2019 | Am J Reprod Immunol | 1 | 1 | 1 | 0 | 1 | 1 | 0 | 6 | V3-V4 |

Table S3: Articles excluded at the last stage and reasoning

| **Articles** | **Reasoning** |
| --- | --- |
| Hyman, R. W., et al. (2014). "Diversity of the Vaginal Microbiome Correlates With Preterm Birth." Reproductive Sciences **21**(1): 32-40.  Nasir, S. A., et al. (2018). "Lactobacillus species detected by 16S rRNA gene sequence isolated from the vaginae of pregnant women and its relation to preterm labor." International Journal of Research in Pharmaceutical Sciences **9**(1): 160-164.  Stout, M., et al. (2014). "The vaginal microbiome of preterm birth." American Journal of Obstetrics and Gynecology **210**(1): S199-S199.  Subramaniam, A., et al. (2016). "Vaginal Microbiota in Pregnancy: Evaluation Based on Vaginal Flora, Birth Outcome, and Race." American Journal of Perinatology **33**(4): 401-408.  Wheeler, S., et al. (2018). "The relationship of cervical microbiota diversity with race and disparities in preterm birth." J Neonatal Perinatal Med **11**(3): 305-310.  Callahan, B. J., et al. (2017). "Replication and refinement of a vaginal microbial signature of preterm birth in two racially distinct cohorts of US women." Proceedings of the National Academy of Sciences of the United States of America **114**(37): 9966-9971.  de Freitas, A. S., et al. (2020). "Defining microbial biomarkers for risk of preterm labor." Brazilian Journal of Microbiology **51**(1): 151-159. | Lack of CST grouping of results |
| Kindinger, L. M., et al. (2017). "The interaction between vaginal microbiota, cervical length, and vaginal progesterone treatment for preterm birth risk." Microbiome **5**. | Received cerclage during pregnancy |
| Hocevar, K., et al. (2019). "Vaginal Microbiome Signature Is Associated With Spontaneous Preterm Delivery." Frontiers in Medicine **6**. | Samples taken after signs of labor |
| Nasioudis, D., et al. (2017). "Influence of Pregnancy History on the Vaginal Microbiome of Pregnant Women in their First Trimester." Scientific Reports **7**. | No information on preterm birth |
| Amabebe, E., et al. (2018). "Mid-gestational changes in cervicovaginal fluid cytokine levels in asymptomatic pregnant women are predictive markers of inflammation-associated spontaneous preterm birth." Journal of Reproductive Immunology **126**: 1-10. | PCR instead of sequencing |
| DiGiulio, D. B., et al. (2015). "Temporal and spatial variation of the human microbiota during pregnancy." Proceedings of the National Academy of Sciences of the United States of America **112**(35): 11060-11065. | Multiple CSTs because of multiple sampling points |

Table S4: Overview and methodological quality of the 17 included studies in the meta-analysis on the vaginal microbiome and the risk of preterm birth (PTB).

| **Study** | **PMID** | **Country** | **Study period** | **Cohort size** | **N preterm births** | **Preterm definition** | **Spontaneous preterm birth** | **Maternal age, in years** | **Ethnicities/Race** | **Gestational week of sampling** | **Sequencing technique** | **Hypervariable region** |
| --- | --- | --- | --- | --- | --- | --- | --- | --- | --- | --- | --- | --- |
| Blostein, 2020 | 31883841 | Peru | Oct 2013 – May 2014 | 125 | 25 (20%) | Before 37 completed gestation weeks | Yes | > 18 | Hispanic | < 16 | 16S | V4 |
| Chang, 2020 | 31838792 | Korea | Not available | 76 | 28 (37%) | Before 37 completed gestation weeks | Yes | 26-41 | Korean | 16-20 | 16S | V1-V3 |
| Dunlop, 2021 | 33996627 | USA | Not available | 359 | 44 (12%) | Before 37 completed gestation weeks | Yes | 18-40 | African American | 8-14 | 16S | V3-V4 |
| Elovitz, 2019 | 30899005 | USA | Dec 2013 – Feb 2017 | 539 | 107 (20%) | Before 37 completed gestation weeks | Yes | Mean 28 | 75% African American 21% White 4% Other | (1) 16-20, (2) 20-24, (3) 24-28 | 16S | V3-V4 |
| Feehily, 2020 | 33184260 | Ireland | Not available | 49 | 8 (16%) | Before 37 completed gestation weeks | Yes | Mean 33 | 88% White Irish 6% White Caucasian  2% Asian  4% African | Second trimester | Shotgun sequencing | NA |
| Fettweis, 2019 | 31142849 | USA | Not available | 135 | 45 (33%) | Before 37 completed gestation weeks | Yes | Mean 26 | 79% African 14% European 6 Hispanic 1% Native American | Average week 18 | 16S | V1-V3 |
| Freitas, 2018 | 29954448 | Canada | Not available | 216 | 46 (21%) | Before 37 completed gestation weeks | Yes | 21-45 | 60% White 15% East Asian 9% South/Southeast Asian 5% Latin American/Hispanic 2% Black 7% Other/mixed ethnicity 1% missing data | 11-16 | 16S | V3 |
| Gudza-Mugabe, 2020 | 31722395 | Zimbabwe | Not available | 244 | 42 (17%) | Before 37 completed gestation weeks | NA | 24-35 | African | 15-35 | 16S | V4 |
| Kindinger, 2017 | 28103952 | United Kingdom | Jan 2013 – Aug 2014 | 161 | 34 (21%) | Before 37 completed gestation weeks | Yes | 21-40 | 65% Caucasian 17% Asian 19% Black | 16 | 16S | V1-V3 |
| Kumar, 2021 | 33747983 | Thailand and Myanmar | Not available | 381 | 19 (5%) | Before 37 completed gestation weeks | Yes | 20-27 | Asian | 8-14 | 16S | V1-V3 |
| Nelson, 2016 | 27057772 | USA | Jul 2008 – Sept 2011 | 40 | 13 (33%) | Before 37 completed gestation weeks | Yes | Mean 19 | African American | <16 or 20-24 | 16S | V4 |
| Odogwu, 2021 | 33504666 | Nigeria | Dec 2018 – Sept 2019 | 38 | 8 (21%) | Before 37 completed gestation weeks | NA | 24-41 | African | 17-21 | 16S | V3-V5 |
| Romero, 2014 | 24987521 | USA | Not available | 90 | 18 (20%) | Before 34 completed gestation weeks | Yes | 20-28 | 88% African American 6% White 6% Others | Monthly up to 24 weeks, then twice a month | 16S | V1-V3 |
| Sarmento, 2021 | 33517811 | Brazil | Not available | 146 | 13 (9%) | Before 37 completed gestation weeks | Yes | Mean 25 | 52% White 39% Mixed race 9% Black | Second trimester | 16S | V1-V3 |
| Stafford, 2017 | 28878691 | United Kingdom | Not available | 134 | 27 (20%) | Before 37 completed gestation weeks | Yes | Mean 29 | 83% White 6% Black 3% Asian 2% Mixed 6% No data | 20-36 | 16S | V1-V3 |
| Tabatabaei, 2019 | 29791775 | Canada | Not available | 450 | 94 (21%) | Before 37 completed gestation weeks | Yes | Not available | 73% White European 6% Black African 1% African American 3% East Asian 1% South Asian 7% Arab 6% South/Central American <1% Canadian aboriginal 3% Other | 8-14 | 16S | V4 |
| You, 2019 | 31134711 | Korea | Not available | 58 | 44 (76%) | Before 37 completed gestation weeks | Yes | Mean 30 | Korean | 22-37 | 16S | V3-V4 |
